# Accelerated *FoxP2* Evolution in Echolocating Bats

**DOI:** 10.1371/journal.pone.0000900

**Published:** 2007-09-19

**Authors:** Gang Li, Jinhong Wang, Stephen J. Rossiter, Gareth Jones, Shuyi Zhang

**Affiliations:** 1 School of Life Science, East China Normal University, Shanghai, China; 2 Institute of Zoology and Graduate University, Chinese Academy of Sciences, Beijing, China; 3 School of Biological and Chemical Sciences, Queen Mary, University of London, London, United Kingdom; 4 School of Biological Sciences, University of Bristol, Bristol, United Kingdom; University of Uppsala, Sweden

## Abstract

*FOXP2* is a transcription factor implicated in the development and neural control of orofacial coordination, particularly with respect to vocalisation. Observations that orthologues show almost no variation across vertebrates yet differ by two amino acids between humans and chimpanzees have led to speculation that recent evolutionary changes might relate to the emergence of language. Echolocating bats face especially challenging sensorimotor demands, using vocal signals for orientation and often for prey capture. To determine whether mutations in the *FoxP2* gene could be associated with echolocation, we sequenced *FoxP2* from echolocating and non-echolocating bats as well as a range of other mammal species. We found that contrary to previous reports, *FoxP2* is not highly conserved across all nonhuman mammals but is extremely diverse in echolocating bats. We detected divergent selection (a change in selective pressure) at *FoxP2* between bats with contrasting sonar systems, suggesting the intriguing possibility of a role for *FoxP2* in the evolution and development of echolocation. We speculate that observed accelerated evolution of *FoxP2* in bats supports a previously proposed function in sensorimotor coordination.

## Introduction

The role of the forkhead transcription factor *FOXP2* in communication was first suggested following the discovery that mutations at this locus cause impaired speech-related motor coordination (orofacial dyspraxia) and comprehension (dysphasia) [1], [2]. Observations that *FOXP2* orthologues show almost no variation across distantly related species of reptile, bird and mammal [3]–[5], while the gene differs by two adaptive amino acid changes between humans and chimpanzees [3], [5], has led to speculation that recent evolutionary changes in *FOXP2* might be related to the emergence of language [3], [5], [6]. More recently, the lack of isolation calls produced by *FoxP2* knockout mice [7], and concordant patterns of expression in the brains of humans, mice [8] and songbirds 9], support a wider function in sensorimotor integration and motor learning [10], [11].

Echolocating bats face especially challenging sensorimotor demands, using vocal signals for orientation and prey capture. They can emit echolocation pulses at rates of up to 200 sounds per second, interpret the resulting echoes within time-windows as short as several milliseconds and make motor responses such as changes in flight manoeuvres during these short time intervals [12]. The reception of ultrasonic pulses for orientation, obstacle avoidance and prey capture in flight require complex aural and either orofacial or, in some species, nasofacial coordination [13]–[15]. Bat echolocation signals show great diversity, contain complex tonal information, and can be modified in response to echo feedback from targets [13], [14]. Auditory processing of echoes in the cochlea and brainstem involve several nuclei that show hypertrophy and differentiation in bats compared with other mammals, including the anteroventral cochlear nucleus, superior olivary complex and the inferior colliculus [16], [17], which appears to show specialisations for echo detection [18]–[20]. Regions of the cerebellum, including the parafloccular lobes and the medial lobe (homologous to vermal lobules VI-VIII in other mammals), also show expansion in echolocating bats compared to non-echolocating mammals [21]. Pulse-echo delay-tuned neurons encoding target distance are present in the cortex and thalamus (medial geniculate body) [22]–[24].

Several of the neural areas implicated in echolocation in bats have also been shown to be associated with *FOXP2*/*FoxP2* expression in the brains of other vertebrate species. Sensory nuclei, including the superior and inferior colliculi, show localised expression in the brains of the adult mouse and human foetus [8], [11], [25], while the thalamus (including the lateral and medial geniculate bodies) and the cerebellum also show localised *FOXP2*/*FoxP2* mRNA expression in the brains of mammals and birds [8], [9], [25], [26]. This overlap, together with the general acceptance that *FOXP2*/*FoxP2* functions in sensorimotor coordination, raises the possibility that *FoxP2* might be involved in the neural circuits that underpin bat echolocation. Furthermore, bats are also one of only a few groups of vertebrates to exhibit vocal learning [27], [28], a condition that might be a precursor to language, and the evolution of which has been linked to developmental and seasonal patterns of *FoxP2* expression in the brains (area X) of some songbirds [9].

Our understanding of the phylogenetic relationships among extant bat species has increased considerably over the past two decades [29]–[36]. Bats diverged from other ordinal groups within the Laurasiatheria around 80 million years ago [37] and diversified in the early Eocene [33]. Bats were traditionally split into the two suborders Microchiroptera (bats that echolocate by producing sounds in the larynx) and Megachiroptera (Old World fruit bats represented by the single family Pteropodidae), however, the former is now known not to represent a true clade. Instead, the emerging and highly resolved molecular tree places all megachiropterans (which do not possess laryngeal echolocation) with some members of the microchiropteran superfamily Rhinolophoidea (horseshoe bats and allies) in a proposed new clade called the Yinpterochiroptera [33], [35], [36]. Other microchiropterans group together in a second clade-the Yangochiroptera-and this new arrangement raises the question of whether laryngeal echolocation has either evolved twice independently or has been lost in the Old World fruit bats [33], [35], [36], [38]. Although similarities between early fossil bats -which appear basal to all other bats-and extant echolocating species appear to support the latter scenario [38], others have argued in favour of convergence in the two clades [32]. A possible loss of laryngeal echolocation appears especially interesting given that one genus of cave roosting fruitbat (*Rousettus*) has subsequently evolved a simple from echolocation based on tongue clicking [38].

Microchiropteran echolocation calls can be broadly classified as either predominately low duty cycle (i.e. signal switched on for typically <20% of the time), frequency modulated (FM) or high duty cycle (>30%) constant frequency (CF) [14]. Most lineages within the suborder Yangochiroptera use orally emitted FM signals, whereas the echolocating Yinpterochiroptera includes CF species that are mostly nasal emitters and able to modify their calls to compensate for Doppler shifted echoes induced by their own flight speed [14], [38]. However, many exceptions exist, and sonar features such as Doppler shift compensation, nasal emission and whispering echolocation, as well as passive listening used by some gleaning species to localize prey, all show independent origins across phylogentically distant groups [38].

To assess whether mutations in the *FoxP2* gene could be associated with echolocation in bats, we sequenced *FoxP2* from echolocating and non-echolocating bat species, as well as a range of other mammals. We isolated mRNA and used reverse transcriptase-mediated polymerase chain reaction (RT–PCR) to amplify the complete gene in bats from six families, as well as representatives from five other mammalian orders and one reptile. Alignments of new sequences with published *FoxP2* sequences from mouse, primates and birds, and those obtained from archived genomic bacterial artificial chromosome (BAC) libraries, revealed high amino acid conservation across most vertebrates [3]–[5] but two highly variable exons in bats. We tested for and found evidence of divergent selection between the two main clades of bats, which have contrasting sonar signals. We also identified two *FoxP2* exons that showed particularly high levels of variability and therefore surveyed these exons in a much wider range of bat species, as well as 18 cetacean species comprising 15 echolocating toothed whales and dolphins (suborder Odontoceti) and three non-echolocating baleen whales (suborder Mysticeti). This extensive survey confirmed that non-synonymous variation among bats exceeds levels recorded across all other vertebrates, but did not suggest that equivalent accelerated evolution was also a feature of echolocating cetaceans.

## Results

### Complete *FoxP2* sequences

We sequenced the complete *FoxP2* gene in 13 bat species, 7 other eutherian mammal species and 1 reptile. After combining with archived sequences, including those obtained from genomic BAC libraries, our analyses of complete *FoxP2* gene sequences were based on 13 bats, 22 additional (non-bat) eutherian mammals, 1 non-eutherian mammal (platypus), two birds and one reptile (see table S1).

**Figure 1:**
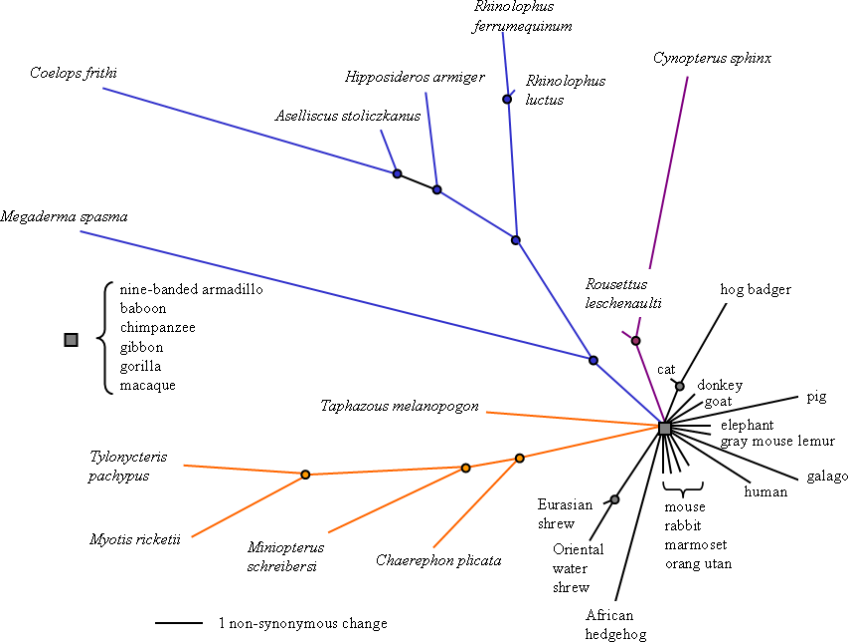
Radial phylogenetic tree showing relative rates of non-synonymous evolution among 35 eutherian mammals, including 13 bats. Bats species are given as italicised binomial names. Branch lengths based on maximum-likelihood estimates of non-synonymous substitutions along 1995 bp of the *FoxP2* gene are superimposed onto a cladogram based on published trees [31], [33], [34]. Bat lineages are coloured to show the echolocating Yinpterochiroptera (blue) that mostly possess high duty constant frequency (CF) calls with at least partial Doppler shift compensation, the Yangochiroptera (orange) that mostly possess low duty cycle calls, as well as the absence of laryngeal echolocation in Yinpterochiroptera fruit bats (violet). The taxa analysed are listed in the Methods and in table S2.

**Figure 2:**
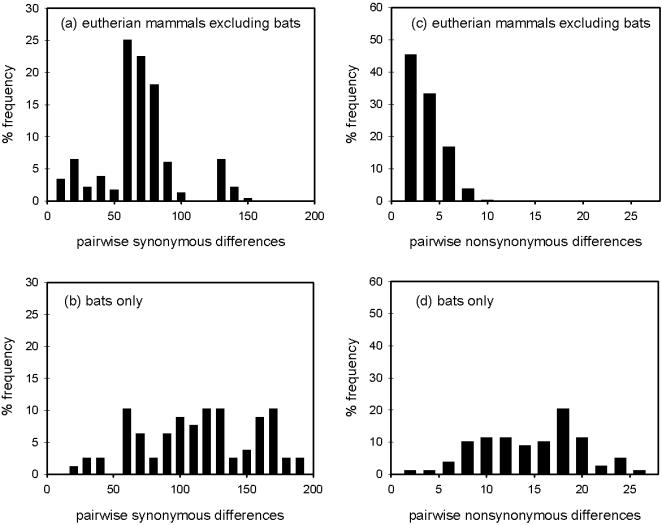
Percentage frequency distributions of pairwise Nei-Gojobori synonymous and non-synonymous differences among 22 eutherian non-bats (a and c, respectively) and among 13 bats (b and d, respectively) based on 1995 bp of *FoxP2*. The taxa analysed are listed in the Methods and in table S2.

**Figure 3:**
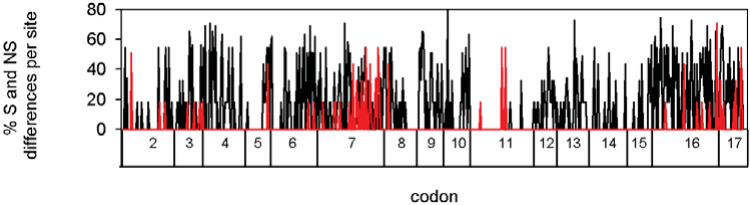
Percentage synonymous (black) and non-synonymous (red) differences per site along the *FoxP2* gene in 13 bat species (listed in table S2). Exon numbers follow [ref. 1].

Alignments revealed that bats show unparalleled numbers of non-synonymous changes compared with other eutherian mammals, (figure 1 and table S2 and S3). An analysis of polymorphic sites in 22 sequences of non-bat eutherian mammals revealed a total number of 365 synonymous changes and 20 non-synonymous changes. By comparison, nearly half the number of bat sequences revealed 385 synonymous changes and more than double (44) the number of non-synonymous changes. We also found significantly greater levels of divergence among bats than among other eutherian mammals at both non-synonymous (mean number of pairwise non-synonymous differences = 13.3 versus 4.3, respectively; Kolmogorov-Smirnov test = 0.88, *P*<0.001) and synonymous sites (mean number of pairwise synonymous differences = 99.4 versus = 78.7, respectively; Kolmogorov-Smirnov test = 0.58, *P*<0.001) (figure 2). The distribution of non-synonymous changes among bats was not uniform along the gene but instead showed peaks around the coding exons 7 and 17 (figure 3).

Branch-Site models detected no sites under positive selection along branches ancestral to all bats, the two major clades of bats or the Yinpterochiroptera excluding fruit bats (results not shown). On the other hand, we found evidence of a change in the strength of selection [39] in *FoxP2* sequences between bats and taxa from related orders within the superorder Laurasiatheria. Significant variation in *ω* was detected with around 16% of sites identified as evolving under divergent selection (likelihood ratio test = 36.3, df = 3, *P*<0.001) (table 1). The higher estimated *ω* for bats over other laurasiatherians (0.23 versus 0.14) reflects the greater number of non-synonymous changes in this group. We repeated the analysis comparing the two major recognised lineages of bats (Yinpterochiroptera and Yangochiroptera) and found evidence for divergent selection at 19% sites (likelihood ratio test = 13.86, df = 3, *P*<0.01) with respective *ω* values of 0.23 and 0.14 (table 1). Thus most change in selective constraints operating on the *FoxP2* protein probably occurred since these lineages diverged. We reconstructed the ancestral amino acid sequences for all bats, for yangochiropterans and for yinpterochiropterans, and confirmed that these are identical to the mammalian consensus sequence as well as the ancestral sequences of the Laurasiatheria and the Euarchontaglires. Both of these comparisons were also significant after fruit bats (Pteropodidae) were excluded from the analyses (for results table 1).

**Table 1:** Parameter estimates and log likelihood values for tests of divergent selection in *FoxP2* sequences among selected groups of mammals.

comparison	model	*ℓ*	parameter estimate	*P*
bats vs. 8 other laurasiatherians	Model C	−7227.29	*ω_0_* = 0, *p_0_* = 0.839	5
			(*ω_1_* = 1), *p_1_* = 0	
			*ω_2_* = 0.139, *ω_3_* = 0.226 (*p_2_* = 0.16)	
	M1a (Nearly Neutral)	−7245.44	*ω_0_* = 0.019, *p_0_* = 0.974	2
			(*ω_1_* = 1, *p_1_* = 0.026)	
Yinpterochiroptera vs. Yangochiroptera	Model C	−5726.79	*ω_0_* = 0, *p_0_* = 0.809	5
			(*ω_1_* = 1), *p_1_* = 0	
			*ω_2_* = 0.137, *ω_3_* = 0.233 (*p_2_* = 0.19)	
	M1a (Nearly Neutral)	−5733.72	*ω_0_* = 0.024, *p_0_* = 0.98	2
			(*ω_1_* = 1, *p_1_* = 0.019)	
bats (excl. fruit bats) vs. 8 other laurasiatherians	Model C	−6967.79	*ω_0_* = 0, *p_0_* = 0.839	5
			(*ω_1_* = 1), *p_1_* = 0	
			*ω_2_* = 0.138, *ω_3_* = 0.204 (*p_2_* = 0.161)	
	M1a (Nearly Neutral)	−6980.43	*ω_0_* = 0.019, *p_0_* = 0.98	2
			(*ω_1_* = 1, *p_1_* = 0.02)	
Yinpterochiroptera (excl. fruit bats) vs. Yangochiroptera	Model C	−5434.08	*ω_0_* = 0, *p_0_* = 0.828	5
			(*ω_1_* = 1), *p_1_* = 0	
			*ω_2_* = 0.155, *ω_3_* = 0.227 (*p_2_* = 0.172)	
	M1a (Nearly Neutral)	−5438.53	*ω_0_* = 0.022, *p_0_* = 0.982	2
			(*ω_1_* = 1, *p_1_* = 0.017)	
fruit bats vs. Yinpterochiroptera (excl. fruit bats)	Model C	−4402.17	*ω_0_* = 0.034, *p_0_* = 0.982	5
			(*ω_1_* = 1), *p_1_* = 0.007	
			*ω_2_* = 0, *ω_3_* = 3.726 (*p_2_* = 0.01)	
	M1a (Nearly Neutral)	−4404.5	*ω_0_* = 0.035, *p_0_* = 0.988	2
			(*ω_1_* = 1, *p_1_* = 0.01216)	

*P* is the number of parameters.

**Table 2:** Likelihood ratio tests for tests of divergent selection in *FoxP2* sequences among selected groups of mammals.

comparison	2Δ*ℓ*	df	*P* value
bats vs. 8 other laurasiatherians	36.3	3	*P*<0.001
Yinpterochiroptera vs. Yangochiroptera	13.86	3	*P*<0.01
bats (excluding fruit bats) vs. 8 other laurasiatherians	25.28	3	*P*<0.001
Yinpterochiroptera (excluding fruit bats) vs. Yangochiroptera	8.9	3	*P*<0.05
fruit bats vs. Yinpterochiroptera (excluding fruit bats)	4.66	3	*P*>0.1

We used a Multivariate Analysis of Protein Polymorphism [40] to predict the physiochemical impact of bat-specific amino acid residues on the *FoxP2* protein structure and found that, on average, the observed changes had less effect than other possible replacements (mean rank of MAPP score for actual replacements = 3.48 versus an expected value of 4.14±0.003 (SE) based on 10,000 randomisations; *Z* test; *P* = 0.02). This finding, together with the absence of stop codons, suggests that overall *FoxP2* remains constrained in bats despite the comparatively high level of variation. However, at the same time, the distribution of predicted impact scores of observed replacements (mean MAPP score = 20.75±1.32 , N = 51) was positively skewed (Kolmogorov-Smirnov test for normality = 0.13, *P* = 0.037) with some changes expected to have a considerable influence on physiochemical properties.

### Survey of *FoxP2* exons 7 and 17

Based on our alignments of complete gene sequences in bats, we identified two highly variable regions (exons 7 and 17). We undertook targeted sequences of these coding regions in a wider range of echolocating and non-echolocating bat species, as well as echolocating and non-echolocating cetaceans and additional eutherian mammals. In total, we obtained exon data for 65 mammal species, including 42 bats (from 10 families) and 18 cetaceans (from two families), two birds and one reptile (table S1).

Alignments of exons 7 and 17 revealed the extent of accelerated amino acid evolution among bat lineages compared with other vertebrates as well as high conservation among vertebrates in general (see tables S4 and S5). At exon 7, although high conservation across non-bats was confirmed [4], we also found previously unreported single amino acid changes in two eulipotyphlan insectivores (Ala283), pig (Asp280) and, the goat (Thr315), though the latter of these was also previously found in the cow [4]. All cetaceans shared three amino acid substitutions (Pro302, Ala304 and Met316) but no differences occurred between echolocating toothed whales and non-echolocating baleen whales. The site Met316 was also recorded in all members of the bat family Hipposideridae within the Yinpterochiroptera, while the Pro302 was shared by all members of the family Vespertilionidae in the Yangochiroptera. Of the two mutations in exon 7 previously linked to language development in humans (Asn303 and Ser325), the former was not identified in any other species, while the latter was found to be shared by the two carnivores sequenced as well as a major clade of bats comprising the echolocating members of the Yinpterochiroptera. Exon 17 was invariant across all non-bat eutherian mammals surveyed with the exception of a single non-synonymous substitution (I697M) in the pig. Within bats, however, amino acid variation was considerable, with between up to 8 nonsynonymous changes recorded for a species (*Nycteris*). Variation at both exons appeared to correspond well to echolocation types/phylogenetic boundaries, with almost complete conservation across groups of confamilial species but contrasting signatures between families.

## Discussion

The *FOXP2*/*FoxP2* gene has been of tremendous interest in recent years, with sequence variation and patterns of expression linked to human speech defects [1], [41], [42], the evolution of language [5], vocal learning in animals [4], [9], [43], and sensorimotor performance more generally [7], [11], [44]. Here we present the most extensive taxonomic survey of *FoxP2* sequences undertaken to date and report that when compared with other groups, this gene shows greater variation in bats.

Although *FoxP2* has clearly undergone accelerated evolution in bats compared to other vertebrates, the basis for this change is not clear. Significant divergent selection based on differences in the *d*_N_/*d*_S_ ratios (*ω*) was detected between laurasiatherians and bats, and also between the two major clades of bats, indicative of a change in the strength of natural selection between these groups. In these tests, higher *d*_N_/*d*_S_ ratios were found in all bats and the clade Yinpterochiroptera, respectively, and values were almost identical, indicating that the detected change in selective pressure has probably occurred since the Yangochiroptera and Yinpterochiroptera diverged. Furthermore, both analyses gave similar results when fruit bats were removed, and thus the divergent selection cannot be attributed to the inclusion of taxa that might have lost the ability (and need) to echolocate.

The higher *d*_N_/*d*_S_ ratio in the Yinpterochiroptera could arise from either a relaxation in selection pressure or a short burst of Darwinian selection. Although explicit tests for positive selection proved negative, the power of such tests to detect positive selection where sequence variability is low is very limited and prone to false negatives (type 2 error) [45]–[47]. It is therefore not possible to rule out the possibility that a period of Darwinian selection has occurred. Indeed several aspects of the system suggest to us that a relaxation in *FoxP2* in echolocating bats is unlikely. First, the fact that *FoxP2* is implicated in sensorimotor coordination in mammals and echolocation bats face exceptional challenges in such coordination provide *a priori* reasons to expect full *FoxP2* functionality in all echolocating bats. Second, *FoxP2* is highly conserved across divergent lineages of vertebrates including birds, reptiles and mammals, and there is no reason why to suspect that *FoxP2* should be any less important in bats than other taxonomic groups. Third, an absence of stop codons, and the non-random distribution of nonsynonymous changes among exons in bat *FoxP2* sequences, which are also predicted to have less impact on the protein's physiocochemical properties than random changes, all suggest that the amino acid replacements are not due to relaxed selection. For these reasons we contend that the changes we report are more likely to have some adaptive significance. The observed correlation between substitution rates at neighbouring non-synonymous and synonymous sites could arise by hitchhiking effects, in which selection increases the fixation probability of linked weakly deleterious sites [48].

One possible explanation for higher *FoxP2* variation in bats might relate to their capacity for vocal learning, which has been described in a number of species [27], [28], [49], [50]. Following speculation that mutations in humans *FoxP2* might be associated with the evolution of language, a number of studies have screened *FoxP2* in vertebrate species that show evidence of vocal learning, which is widely considered to be a critical substrate for the evolution of human language. In addition to bats, vocal learning has also been reported in some lineages of birds [51]–[55], cetaceans [56]–[58] (see ref. [59] for a review) and was recently described in the African elephant [60]. Evidence from *FoxP2* expression studies in avian brains have proven inconclusive [9], and sequence comparisons between non-vocal learners and song-learning birds as well as a small number of vocal learning mammals, have not identified specific mutations related to this ability [4], [9], [11]. The current study, which represents a considerable increase in the number of vocal learning taxa considered, with, 42 bat species, 18 cetacean species and African elephant added, found no consistently shared non-synonymous mutations among these species. Our results thus appear to support earlier studies that found no evidence of specific mutations associated with vocal learning abilities [3], [4], though we cannot rule out differential expression of this gene. Indeed, though some related bats in the Yinpterochiroptera were found to share a Ser325 residue with humans, the absence of this amino acid in other bat lineages, together with its presence in carnivores (also described previously [3]), suggest that it is unlikely to be associated with vocal learning. Perhaps more interesting is the result that two out of three amino acid substitutions that have occurred in the cetaceans (Pro302 and Met316) have also arisen independently in some bat families. These changes could in theory be linked to the evolution of vocal learning, however, if this were the case, then their absence in other bat genera known to exhibit learning (e.g. *Rhinolophus*) and the fact that both changes do not occur together in any bat, mean that if variation in *FoxP2* has a role in vocal learning then it is not straightforward.

We instead speculate that observed variation in bats might be associated with aspects of echolocation. As mentioned, amino acid variation at both exons 7 and 17 in bats corresponds well to echolocation types/phylogenetic boundaries, with almost complete conservation across groups of confamilial species but contrasting signatures between families. Such high sequence diversity at exons 7 and 17 in bats relative to other mammals, including echolocating cetaceans, indicates that *FoxP2* plays a role in the sensorimotor demands that are peculiar to bat echolocation rather than echolocation in general. Indeed while all cetaceans shared three amino acid substitutions (Pro302, Ala304 and Met316) no differences were observed between echolocating and non-echolocating baleen cetaceans, also supporting an earlier comparison of one example from each suborder [4].

The absence of consistent shared amino acids across echolocating bats and cetaceans is not necessarily inconsistent with our hypothesis that *FoxP2* variation is linked to echolocation in bats. *FoxP2* has previously been implicated in sensorimotor disorders with respect to vocalisations and orofacial coordination [1], [7], [44] and, although whales do show complex vocal behaviour, their sonar signals are emitted through their forehead (melon), thus circumventing the need for such rapid orofacial coordination. Moreover, echolocating whales and dolphins use relatively stereotyped clicks, and show neither the signal variability of bats nor the dynamic modification of signal design in relation to echo feedback [61].

A lack of clear differences in nonsynonymous variation at exon 7 between echolocating and non-echolocating lineages in both cetaceans and bats can also be explained if the common ancestor in both groups had echolocation and this ability was subsequently lost in some lineages. This scenario has been previously been suggested for bats [36], though some disagree [32]. On the other hand, if *FoxP2* does function in echolocation, then the signature at exon 17 might be more suggestive that echolocation has evolved twice in bats. Here, fruit bats that do not have laryngeal echolocation (Yinpterochiroptera) (echolocation status 1 and 2 in tables S2, S3 S4 and S5) show no differences from the dominant eutherian mammal sequence, while two amino acid changes are present in both the related genera *Rhinolophus* (T641A and D663E) and *Hipposideros* (V637E and D663E), with further non-synonymous substitutions in the hipposiderid genera *Aselliscus* (I602M and E657D) and *Coelops* (I621V, N644D, I655M and E657D). In cetaceans, evidence regarding the evolution of echolocation is also equivocal but fossil evidence indicates that early species were toothed rather than possessing baleen and could hear underwater, though there is no evidence to date of anatomical specialisations associated with echolocation [62], [63].

Trends in *FoxP2* sequence variation with respect to other cases of convergence in bat echolocation are also informative. The methionine to isoleucine substitution (M637I) in *Hipposideros* has also arisen independently in the yangochiropteran species *Pteronotus parnellii* but not in its congeners *P. quadridens* or *P. macleayii*. It is therefore interesting that *P. parnellii* is the only species of non-rhinolophoid bat to have evolved Doppler-shift compensation in echolocation, a feature that it shares with the genus *Hipposideros*. A striking example of rapid *FoxP2* evolution is that of the yangochiropteran genus *Nycteris*, which, unlike its closest surveyed relatives, shows independent evolution of nasal emission, multi-harmonic call structure and prey location by passive listening [38], [64]. *Nycteris* also shows the highest rate of non-synonymous change at exon 17 of all species surveyed, differing from both the mammalian consensus and its closest relative by eight amino acids (V678A, A680T, T692A, E698A, L699F, D701E, I705E, L710S; table S4). Prior to this study, data on *FoxP2* in bats was limited to a partial sequence of exon 7 in a single individual (*Tadarida sp*., family Molossidae) [3], [4]. Two amino acid differences at exon 7 (A307V and L292S) between the published *Tadarida* sequence and its close relative *Chaerephon plicata* (family Molossidae) further highlight the high degree of non-synonymous diversity in this order.

Comparatively high levels of *FoxP2* sequence diversity in bats presented in this study, together with evidence of significant divergent selection and a lack of evidence of positive selection at this time, all point to a need for more work to elucidate the role of this protein in bats. Based on current findings, we suggest that echolocation in bats might have involved the recruitment of the *FoxP2*, and, more generally, that the protein might function in the mobilisation of downstream genes (or genetic cascades) involved in one or more aspects of the development or regulation of complex sensorimotor coordination. To investigate these speculations, data on the expression of *FoxP2* in the bat brain, with particular attention to the neural pathways implicated in echolocation, would allow informative comparisons with published results from human, mouse and bird brains. To date, the delineated *FoxP2* protein domains thought to be involved in DNA binding and transcription regulation [65] do not include either exon 7 or exon 17, though acidic transcription activation domains have been identified in the C-terminal of other forkhead genes [66]. Therefore the characterisation of these comparably variable domains in bats and other species, the former of which also contains two amino acids implicated in the evolution of human language [5], should also to help assess further the potential role and evolutionary significance of *FoxP2* in sensorimotor integration and rapid motor learning.

## Materials and Methods

### Sample collection

Bat wing membrane biopsies were collected from five localities in China. Some individuals were sacrificed as part of an ongoing surveillance programme for coronaviruses, and their liver and brain tissue stored in liquid nitrogen for RNA preservation. Additional genetic material from bats and other mammals was obtained from tissue banks held at the Institute of Zoology in Beijing and the Institute of Zoology in London.

### Isolation, amplification and sequencing of nucleic acids

We used RT-PCR to amplify the complete *FoxP2* coding sequence from mRNA transcripts. Total RNA was isolated from brain and liver tissue using RNAiso kits (TaKaRa, Japan) and reverse-transcribed to first-strand cDNA using Invitrogen SuperScript™ II RT and oligo-dT primers. We designed two primers pairs based on conserved sections to amplify two overlapping regions, spanning exons 2 to 10 (5′-GGT ATT AAG TCA TGA TGC AGG A-3′ and 5′-TCG CAT GTG CAA GTG GGT C-3′) and exons 8 to 17 (5′-GGC TGT GAA AGC ATT TGT GAA G-3′ and 5′-ATG GTT GTG GAG TGG TTA TGA AG-3′). Due to technical problems with amplifying the second region in one species (*Megaderma spasma*), we also designed the degenerate primer 5′-GAG GTY KCA CAA GYC AGT TCT CAT TCC -3′ to work with primer 5′-GGC TGT GAA AGC ATT TGT GAA G-3′.

PCR products from cDNA were ligated into a pMD19-T vector (TaKaRa) and cloned. Positive clones were sequenced using Big Dye Terminator on an ABI 3730 DNA sequencer (Applied Biosystems). Complete sequences were reconstructed by overlapping shorter sequences, allowing us to verify that nucleotide changes were not attributable to RT-PCR artefacts.

From alignments of whole *FoxP2* gene sequences, we identified two regions of especially high variability that correspond to exon 7 (188 bp) and exon 17 (145 bp). To survey these exons in a wider range of bat species, DNA was isolated with DNeasy kits (Qiagen) for PCR-based targeted amplification. For exon 7, we designed the degenerate primer 5′-CTG GCT TAA GTC CTG CYG ARA TTC-3′ to use with the published intronic primer 5′-GAA TAA AGC TCA TGA GAT TTA CCT GTC-3′ [4]. For exon 17, we designed and used three primer pairs (5′-CCA CTT CCC CAT CAC TCT GTT G-3′ and 5′-ATG GTT GTG GAG TGG TTA TGA AG-3′, 5′-CTC TAA CCA GCT CAT GCA ATC-3′ and 5′-ATG GTT GTG GAG TGG TTA TG–3′, 5′-ACT GCT GGG CTG AAG TTG ATT A-3′ and 5′-ATG GTT GTG GAG TGG TTA TG-3′) because no single pair was able to amplify every species. Details of primer combinations are available on request.

### Taxonomic coverage for whole *FoxP2* gene comparsion

For whole gene alignments, we sequenced 2100–2200 bp of the *FoxP2* gene in one individual each of thirteen species of bat (Pteropodidae: *Rousettus leschenaulti*, *Cynopterus sphinx*; Megadermatidae: *Megaderma spasma*; Rhinolophidae: *Rhinolophus ferrumequinum*, *R. luctus*; Hipposideridae: *Hipposideros armiger*, *Aselliscus stoliczkanus*, *Coelops frithii*; Emballonuridae: *Taphozous melanopogon*; Vespertilionidae: *Miniopterus schreibersi*, *Myotis ricketti*, *Tylonycteris pachypus*; Molossidae: *Chaerephon plicata*) as well as pig (*Sus scrofa*), goat (*Capra hircus*), donkey (*Equus asinus*), cat (*Felis catus*), hog-badger (*Arctonyx collaris*), oriental water shrew (*Chimarrogale himalayica*), rabbit (*Oryctolagus cuniculus*) and one reptile species, the red-eared slider terrapin (*Trachemys scripta*).

We also obtained from GenBank the published whole *FoxP2* sequences of six primate species (human (*Homo sapiens*; NM_148898), chimpanzee (*Pan troglodytes*; NM_001009020), gorilla (Gorilla gorilla; AF512948), orang utan (*Pongo pygmaeus*; AH011319), white-handed gibbon (*Hylobates lar*; AH011317), rhesus macaque (*Macaca mulatta*; AF512950) two bird species (zebra finch (*Taeniopygia guttata*, AY395709) and budgerigar (*Melopsittacus undulatus*; AY466101)) and mouse (*Mus musculus*; NM053242). We obtained additional *FoxP2* sequences by searching archived genomic BAC libraries (National Center for Biotechnology Information) using the BLASTN tool (http://www.ncbi.nlm.nih.gov/BLAST/) for olive baboon (*Papio anubis*; AC155878, AC149459, AC149861, AC157859), common marmoset (*Callithrix jacchus*; AC151545, AC151033, AC151040), small-eared galago (*Otolemur garnettii*; AC148960, AC148947, AC151626), gray mouse lemur (*Microcebus murinus*; AC186909, AC185373, AC187418), African elephant (*Loxondonta africana*; AC163970, AC164945, AC164509, AC172736), nine-banded armadillo (*Dasypus novemcinctus*; AC162148, AC152481, AC152132, AC152372, AC152126), African hedgehog (*Atelerix albiventris*; AC187948, AC183856, AC175228, AC173449, AC186110), common shew (*Sorex araneus*; AC168041, AC169146, AC168969, AC168968), and one non-eutherian mammal species, the platypus (*Ornithorhynchus anatinus*; AC155098, AC158426, AC154065).

### Taxonomic coverage for exon 7 and exon 17 comparsion

In addition to sequencing the complete *FoxP2* gene in 13 bat species, we also sequenced exons 7 (188 bp) and 17 (145 bp) in a wider range of bats (Pteropodidae: *Eonycteris spelaea*, *Nyctimene cephalotes*, *Pteropus rodricensis*; Rhinolophidae: *Rhinolophus affinis*, *R. macrotis*, *R. marshalli*, *R. osgoodi*, *R. pearsonii*, *R. pusillus*, *R. paradoxolophus*, *R. sinicus*; Hipposideridae: *Aselliscus tricuspidatus*, *Hipposideros larvatus*, *H. pomona*, *H. pratti*; Megadermatidae: *Megaderma lyra*; Nycteridae: *Nycteris tragata*; Phyllostomidae: *Carollia perspicillata*; Mormoopidae: *Pteronotus macleayii*, *P. parnellii*, *P. quadridens*; Vespertilionidae: *Barbastella leucomelas*, *Ia io*, *Murina* sp., *Nyctalus velutinus*, *Pipistrellus abramus*, *Plecotus* sp., *Scotophilus kuhlii*, *Scotomanes ornatus*, *Vespertilio sinensis*). For 12 bat species (*Cynopterus sphinx*, *Rousettus leschenaulti*, *Rhinolophus pusillus*, *R. ferrumequinum*, *Hipposideros pomona*, *H. amiger*, *Coelops frithi*, *Megaderma lyra*, *M. spasma*, *Nycteris tragata*, *Myotis ricketti* and *Taphozous melanopogon*) DNA was sequenced from multiple individuals (2-5) to confirm the results and in all cases no variation was found between conspecifics.

In addition, to determine whether rapid *FoxP2* evolution is a common feature of vertebrate echolocation, we also sequenced 15 echolocating toothed whales (Atlantic white-sided dolphin (*Lagenorhynchus acutus*), Blainville's beaked whale (*Mesoplodon densirostris*), bottlenose dolphin (*Tursiops truncatus*), common dolphin (*Delphinus delphis*), Cuvieŕs beaked whale (*Ziphius cavirostris*), harbour porpoise (*Phocoena phocoena*), killer whale (*Orcinus orca*), long-finned pilot whale (*Globicephala melas*), northern bottlenose whale (*Hyperoodon ampullatus*), pygmy sperm whale (*Megaptera novaeangliae*), Risso's dolphin (*Grampus griseus*), Sowerby's beaked whale (*Mesoplodon bidens*), sperm whale (*Physeter catodon*), striped dolphin (*Stenella coeruleoalba*) and white-beaked dolphin (*Lagenorhynchus albirostris*) as well as three species of non-echolocating baleen whale (fin whale (*Balaenoptera physalus*), humpback whale (*Megaptera novaeangliae*) and minke whale (*Balaenoptera acutorostrata*)). To improve taxonomic coverage further, we also included the red and white giant squirrel (*Petaurista alborufus*: Rodentia) and the short-eared elephant-shrew (*Macroscelides proboscideus*: Macroscelidea).

GenBank accession numbers of all new complete *FoxP2* sequences are EU076391-EU076411 inclusive. For exons 7 they EU076412 and EU087927-EU087965 inclusive, and for exon 17 they are EU087966-EU088011 inclusive. Information on the taxonomic groupings of all sequenced species is given in tables S2, S3, S4 and S5.

### Statistical analyses

Nucleotide sequences (1995 bp) were aligned using the software ClustalX [67], after removing a polyglutamine stretch [5], which shows length variability in bats and carnivores versus other taxa, and were translated in MEGA 3.1 [68].

For each pair of complete sequences, we derived the total number of Nei-Gojobori synonymous and nonsynonymous differences [69] using the software DnaSP 4.0 [70]. We generated distributions of values of pairwise synonymous and non-synonymous differences for bats and other eutherian mammals and tested for differences between the distributions using a Two-Sample Kolmogorov-Smirnov test in the software S-PLUS 2000 (MathSoft Inc.).

We constructed a phylogeny based on published studies [31], [33], [34] and used the software PAML [71] to derive maximum-likelihood estimates of the number of synonymous and non-synonymous substitutions per site (*d*_S_ and *d*_N_) for each branch assuming independent *d*_S_/*d*_N_ (*ω*) ratios per lineage [72] (see figure S1). We then constructed a radial tree based on *d*_N_ values using the software PHYLODRAW [73].

We tested for evidence of positive selection along selected lineages using Zhang *et al*'s modified Branch-Site Model A [74]. In this model, the phylogeny is partitioned into foreground and background branches, with positive selection potentially occurring along the former. Four site classes of codon are assumed, and two of these, evolving under purifying selection (0<*ω*_0_<1) and neutral selection (*ω*_1_ *= *1), occur across the tree. Two additional sites evolve under purifying or neutral selection on the background branches but under positive selection (*ω*_2_>1) on the branch under consideration (foreground). A likelihood ratio test (LRT) was conducted by comparing the likelihood result of this model to a null model in which all parameters were the same except that *ω*_2 _is fixed at 1 (neutral evolution) on the foreground. We repeated this analysis four times, to test for positive selection along branches ancestral to all bats, to all Yangochiroptera, to all Yinpterochiroptera and to all fruit bats.

If *FoxP2* has in some way become recruited into neural pathways involved in bat echolocation, it might be associated with a change in selective pressure between echolocating bats and other mammals, and also between bat lineages that exhibit contrasting forms of echolocation system. Such changes in selection pressure can be small and detectable even when specific tests of positive selection prove negative. To test for evidence of divergent selective pressures among selected pairs of clades, we applied the modified version of Bielawski and Yang's maximum likelihood-based Clade Model C [39], [71], which is suited to sequence data with low divergence. This model is based on a branch-site model and allows variation in the *d*_S_/*d*_N_ (*ω*) ratio among sites with a proportion of sites evolving under different selective constraints between a pair of clades [39]. In the modified Clade Model C, the two clades were assumed to share sites under purifying selection (0<*ω*_0_<1) and neutral evolution (*ω*_1_ *= *1), but to differ at a third site class under divergent selection (*ω*_2__clade 1_≠*ω*_2 clade 2_). We compared the model results with those obtained from the site model M1a (Nearly Neutral) to conduct a LRT with 3 degrees of freedom.

We first test for evidence of divergent selection (i.e. a change in the strength of natural selection) between bats and their nearest known relatives in the superorder Laurasiatheria, represented by a clade comprising donkey, pig, goat, shrew, Asian shrew, hedgehog, cat and badger. We then compared the two major recognised lineages of bats (Yinpterochiroptera and Yangochiroptera). Both tests were repeated but with fruit bat sequences excluded, to assess whether any changes in selection pressure are likely to be due to relaxation resulting from a loss of echolocation. For the same reason we directly compared fruit bats and non-fruit bats within the clade Yinpterochiroptera. We tested the robustness of our results and tested for suboptimal likelihood peaks by rerunning the model under a range of initial values of *ω*_0, _and found that estimates of the proportion of sites under divergent selection were consistent_._

We also undertook a Multivariate Analysis of Protein Polymorphism (MAPP) [40] to test whether amino acid variants in bats are likely to have had a greater or lower physiocochemical impact on the *FoxP2* protein than random non-synonymous changes. Amino acid sequences of *FoxP2* orthologues of all mammals excluding bats, birds and reptiles were aligned and, to correct for phylogenetic affiliations, were weighted based on PAML-derived branch lengths and known phylogeny topology [40]. From the evolutionary variation present, the physiocochemical constraint of each position was calculated based on several properties (hydropathy, polarity, charge, volume and free energy in an alpha helix and beta sheet), and the predicted impact (MAPP score) of all possible variants assessed [40]. For a second alignment, comprising bat orthologues, the MAPP score of each amino acid variant was derived, together with the score of all other variants at the same site in the alignment that differed from the ancestral sequence by an equal or fewer number of nucleotide substitutions. All such MAPP scores were then sorted and ranked. We then compared the mean MAPP rank score of observed variants to a null distribution of 10,000 mean rank scores, generated using the software MATHEMATICA (Wolfram Research Inc.) from ranks selected at random from the variable sites. Statistical difference between the means was assessed using a Z test.

## Supporting Information

Table S1 Summary of sequences surveyed in the study. Accession numbers are given for sequences obtained from GenBank.
(0.17 MB DOC)

Table S2 *FoxP2* gene (first half) with variable sites shown. For all species represented in tables S2, S3, S4 and S5, abbreviations are given to denote Superordinal (S) group (E (Euarchontaglires), A (Atlantogenata) and L (Laurasiatheria)) and ordinal (O) group (Pr (Primates), Eu (Eulipotyphla), R (Rodentia), L (Lagomorpha), X (Xenarthra), Pr (Proboscidea), M (Macroscelidea), Ar (Artiodatyla), Pe (Perissodactyla), Ca (Carnivora), Ce (Cetacea), Ch (Chiroptera/bats)). Within bats, abbreviations are given to denote family (P = Pteropodidae/fruitbats, R = Rhinolophidae, H = Hipposideridae, Me = Megadermatidae, E = Emballonuridae, M = Molossidae, V = Verspertilionidae, Mo = Mormoopidae, N = Nycteridae, Phyllostomidae) and each family code is followed by either Yi or Ya to denote membership of the newly recognised clade Yinpterochiroptera or Yangochiroptera, respectively. For bats, echolocation status [2] is indicated by superscripts, numbered as follows: 1 = brief broadband tongue clicks (no laryngeal echolocation), 2 = no echolocation, 3 = constant frequency, 4 = short, broadband, multiharmonic, 5 = narrowband, multiharmonic, 6 = narrowband, dominated by fundamental harmonic, 7 = short, broadband, dominated by fundamental harmonic. Residue numbers are based on the human *FOXP2* orthologue. Amino acids that were linked to the evolution of language are given in red and those only found in bats are given in blue.
(0.18 MB DOC)

Table S3 *FoxP2* gene (second half) with variable sites shown. For abbreviations, see Table S2.
(0.17 MB DOC)

Table S4 Exon 7 of *FoxP2*. For abbreviations, see Table S2.
(0.13 MB DOC)

Table S5 Exon 17 of *FoxP2*. For abbreviations, see Table S2.
(0.10 MB DOC)

Figure S1 Cladogram showing PAML-derived maximum likelihood estimates of rates of non-synonymous and synonymous substitutions. Bat lineages are coloured to show the two major divergent forms of laryngeal echolocation (blue = high duty constant frequency (CF) calls with at least partial Doppler shift compensation, orange = low duty cycle calls, green = short, broadband multi-harmonic, violet = the absence of laryngeal echolocation in fruit bats. Estimates of the number of non-synonymous substitutions (dN) and synonymous substitutions (dS) under a free ratio model are given on the branches as (dN/dS).
(0.05 MB DOC)
