# Supplementary material for: Accelerated FoxP2 Evolution in Echolocating Bats
Source: PLoS One. 2007 Sep 19;2(9):e900. doi: 10.1371/journal.pone.0000900 (PMC1976393; doi:10.1371/journal.pone.0000900)
Supplement: Table S1 — Summary of sequences surveyed in the study. Accession numbers are given for sequences obtained from GenBank. (0.17 MB DOC) [file pone.0000900.s001.doc]

| **species** | **family** | **order** | **complete sequence** | **exon 7** | **exon 17** |
| --- | --- | --- | --- | --- | --- |
| **Non-bats mammals** |  |  |  |  |  |
| Human (*Homo sapiens*) | Hominidae | Primates | NM_014491 [1] | NM_014491[1] | NM_014491[1] |
| gorilla (*Gorilla gorilla*) | Hominidae | Primates | AF512948 [5] | AF512948 [5] | AF512948 [5] |
| chimp (*Pan troglodytes*) | Hominidae | Primates | NM_001009020 [5] | NM_001009020[5] | NM_001009020 [5] |
| orang utan (*Pongo pygmaeus*) | Hominidae | Primates | AY143181 [3] | AY143181 [3] | AY143181 [3] |
| gibbon (*Hylobates lar*) | Hylobatidae | Primates | AH011317 [Unpublished] | AH011317 | AH011317 |
| macaque (*Macaca mulatta*) | Cercopithecidae | Primates | NM_001033021[5] | NM_001033021[5] | NM_001033021 [5] |
| baboon (*Papio anubis*) | Cercopithecidae | Primates | AC155878, AC149459, AC149861, AC157859 | AC149861 | AC149861 |
| common marmoset (*Callithrix jacchus*) | Callithricidae | Primates | AC151545, AC151033, AC151040 | AC151033 | AC151033 |
| galago (*Otolemur garnettii*) | Galagidae | Primates | AC148947,AC149244, AC151626 | AC149244 | AC149244 |
| gray mouse lemur (*Microcebus murinus*) | Cheirogaleidae | Primates | AC186909, AC185373, AC187418 | AC185373 | AC187418 |
| Eurasian shrew (*Sorex araneus*) | Soricidae | Insectivora | AC168041, AC169146, AC168969, AC168968 | AC168969 | AC168968 |
| African hedgehog (*Atelerix albiventris*) | Erinaceidae | Insectivora | AC171760, AC173936, AC173449, AC175228, AC183856 | AC173936 | AC173449 |
| Oriental water shrew (*Chimarrogale himalayica*) | Soricidae | Insectivora | This study | This study | This study |
| mouse (*Mus musculus*) | Muridae | Rodentia | NM_053242 [5] | NM_053242 [5] | NM_053242 [5] |
| white giant squirrel (*Petaurista alborufus)* | Sciuridae | Rodentia | n/a | n/a | This study |
| nine-banded armadillo (*Dasypus novemcinctus*) | Dasypodidae | Cingulata | AC162148, AC152481, AC152132, AC152372, AC152126 | AC152372 | AC152126 |
| African elephant (*Loxondonta africana*) | Elephantidae | Proboscidea | AC163970, AC164945, AC164509, AC172736 | AC164509 | AC172736 |
| rabbit (*Oryctolagus cuniculus*) | Lagomorpha | Leporidae | This study | This study | This study |
| pig (*Sus scrofa*) | Suidae | Artiodactyla | This study | This study | This study |
| fin whale (*Balaenoptera physalus*) | Balaenopteridae | Cetacea | n/a | This study | This study |
| minke whale (*B. acutorostrata*) | Balaenopteridae | Cetacea | n/a | This study | This study |
| humpback whale (*Megaptera novaeangliae*) | Balaenopteridae | Cetacea | n/a | This study | This study |
| Atlantic white-sided dolphin (*Lagenorhynchus acutus*) | Delphinidae | Cetacea | n/a | This study | This study |
| white-beaked dolphin (*Lagenorhynchus albirostris*) | Delphinidae | Cetacea | n/a | This study | This study |
| bottlenose dolphin (*Tursiops truncates*) | Delphinidae | Cetacea | n/a | This study | n/a |
| common dolphin (*Delphinus delphis*) | Delphinidae | Cetacea | n/a | This study | This study |
| killer whale (*Orcinus orca*) | Delphinidae | Cetacea | n/a | This study | This study |
| long-finned pilot whale (*Globicephala melas*) | Delphinidae | Cetacea | n/a | This study | n/a |
| Risso's dolphin (*Grampus griseus*) | Delphinidae | Cetacea | n/a | This study | This study |
| striped dolphin (*Stenella coeruleoalba*) | Delphinidae | Cetacea | n/a | This study | This study |
| harbour porpoise (*Phocoena phocoena*) | Phocoenidae | Cetacea | n/a | This study | This study |
| pygmy sperm whale (*Kogia breviceps*) | Physeteridae | Cetacea | n/a | This study | This study |
| sperm whale (*Physeter catodon*) | Physeteridae | Cetacea | n/a | This study | n/a |
| Blainville's beaked whale(*Mesoplodon densirostris*) | Physeteridae | Cetacea | n/a | This study | This study |
| Sowerby's beaked whale (*M. bidens*) | Ziphiidae | Cetacea | n/a | This study | This study |
| Cuvier′s beaked whale (*Ziphius cavirostris*) | Ziphiidae | Cetacea | n/a | This study | This study |
| northern bottlenose whale (*Hyperoodon ampullatus*) | Ziphiidae | Cetacea | n/a | This study | This study |
| goat (*Capra hircus*) | Bovidae | Artiodactyla | This study | This study | This study |
| donkey (*Equus asinus*) | Equidae | Perissodactyla | This study | This study | This study |
| hog badger (*Arctonyx collaris*) | Mustelidae | Carnivora | This study | This study | This study |
| cat (*Felis catus*) | Felidae | Carnivora | This study | This study | This study |
| short-eared elephant-shrew (*Macroscelides proboscideus*) | Macroscelididae | Macroscelidea | n/a | n/a | This study |
| platypus (*Ornithorhynchus anatinus)* | Ornithorhynchidae | Monotremata | AC155098, AC158426, AC154065 | AC154065 | AC154065 |
| **Bats** |  |  |  |  |  |
| *Rousettus leschenaulti* | Pteropodidae | Chiroptera | This study | This study | This study |
| *Pteropus rodricensis* | Pteropodidae | Chiroptera | n/a | This study | This study |
| *Nyctimene cephalotes* | Pteropodidae | Chiroptera | n/a | n/a | This study |
| *Eonycteris spelaea* | Pteropodidae | Chiroptera | n/a | This study | This study |
| *Cynopterus sphinx* | Pteropodidae | Chiroptera | This study | This study | This study |
| *Rhinolophus affinis* | Rhinolophidae | Chiroptera | n/a | n/a | This study |
| *R. ferrumequinun* | Rhinolophidae | Chiroptera | This study | This study | This study |
| *R. luctus* | Rhinolophidae | Chiroptera | This study | This study | This study |
| *R. macrotis* | Rhinolophidae | Chiroptera | n/a | n/a | This study |
| *R. marshalli* | Rhinolophidae | Chiroptera | n/a | This study | n/a |
| *R. osgoodi* | Rhinolophidae | Chiroptera | n/a | n/a | This study |
| *R. paradoxolophus* | Rhinolophidae | Chiroptera | n/a | This study | This study |
| *R. pearsonii* | Rhinolophidae | Chiroptera | n/a | n/a | This study |
| *R. pusillus* | Rhinolophidae | Chiroptera | n/a | This study | This study |
| *Aselliscus stoliczkanus* | Hipposideridae | Chiroptera | This study | This study | This study |
| *A. tricuspidatus* | Hipposideridae | Chiroptera | This study | This study | This study |
| *Coelops frithi* | Hipposideridae | Chiroptera | This study | This study | This study |
| *Hipposideros armiger* | Hipposideridae | Chiroptera | This study | This study | This study |
| *H. larvatus* | Hipposideridae | Chiroptera | n/a | This study | This study |
| *H. pomona* | Hipposideridae | Chiroptera | n/a | This study | This study |
| *H. pratti* | Hipposideridae | Chiroptera | n/a | n/a | This study |
| *Megaderma lyra* | Megadermatidae | Chiroptera | n/a | This study | n/a |
| *M. spasma* | Megadermatidae | Chiroptera | This study | This study | This study |
| *Taphozous melanopogon* | Emballonuridae | Chiroptera | This study | This study | This study |
| *Nycteris tragata* | Nycteridae | Chiroptera | n/a | n/a | This study |
| *Carollia perspicillata* | Phyllostomidae | Chiroptera | n/a | This study | This study |
| *Mormoops blainvillii* | Mormoopidae | Chiroptera | n/a | n/a | This study |
| *Pteronotus parnellii* | Mormoopidae | Chiroptera | n/a | This study | This study |
| *P. macleayii* | Mormoopidae | Chiroptera | n/a | This study | This study |
| *P. quadridens* | Mormoopidae | Chiroptera | n/a | This study | This study |
| *Miniopterus schreibersi* | Vespertilionidae | Chiroptera | This study | This study | This study |
| *Barbastella leucomelas* | Vespertilionidae | Chiroptera | n/a | This study | This study |
| *Ia io* | Vespertilionidae | Chiroptera | n/a | This study | This study |
| *Murina sp.* | Vespertilionidae | Chiroptera | n/a | This study | This study |
| *Myotis ricketti* | Vespertilionidae | Chiroptera | This study | This study | This study |
| *Nyctalus velutinus* | Vespertilionidae | Chiroptera | n/a | n/a | This study |
| *Pipistrellus abramus* | Vespertilionidae | Chiroptera | n/a | This study | n/a |
| *Plecotus sp.* | Vespertilionidae | Chiroptera | n/a | This study | This study |
| *Scotomanes ornatus* | Vespertilionidae | Chiroptera | n/a | This study | This study |
| *Scotophilus kuhlii* | Vespertilionidae | Chiroptera | n/a | This study | This study |
| *Tylonycteris pachypus* | Vespertilionidae | Chiroptera | This study | This study | This study |
| *Vespertilio sinensis* | Vespertilionidae | Chiroptera | n/a | n/a | This study |
| *Chaerephon plicata* | Molossidae | Chiroptera | This study | This study | This study |
| **Non-mammals** |  |  |  |  |  |
| zebra finch (*Taeniopygia guttata*) | Estrildidae | Passeriformes | AY395709 [11] | AY395709 [11] | AY395709 [11] |
| budgerigar (*Melopsittacus undulatus*) | Psittacidae | Psittaciformes | AY466101 [Unpublished] | AY466101 | AY466101 |
| red-eared slider terrapin (*Trachemys scripta*) | Emydidae | Testudines | This study | This study | This study |
