# Supplementary material for: Accelerated FoxP2 Evolution in Echolocating Bats
Source: PLoS One. 2007 Sep 19;2(9):e900. doi: 10.1371/journal.pone.0000900 (PMC1976393; doi:10.1371/journal.pone.0000900)
Supplement: Table S2 — FoxP2 gene (first half) with variable sites shown. For all species represented in tables S2, S3, S4 and S5, abbreviations are given to denote Superordinal (S) group (E (Euarchontaglires), A (Atlantogenata) and L (Laurasiatheria)) and ordinal (O) group (Pr (Primates), Eu (Eulipotyphla), R (Rodentia), L (Lagomorpha), X (Xenarthra), Pr (Proboscidea), M (Macroscelidea), Ar (Artiodatyla), Pe (Perissodactyla), Ca (Carnivora), Ce (Cetacea), Ch (Chiroptera/bats)). Within bats, abbreviations are given to denote family (P = Pteropodidae/fruitbats, R = Rhinolophidae, H = Hipposideridae, Me = Megadermatidae, E = Emballonuridae, M = Molossidae, V = Verspertilionidae, Mo = Mormoopidae, N = Nycteridae, Phyllostomidae) and each family code is followed by either Yi or Ya to denote membership of the newly recognised clade Yinpterochiroptera or Yangochiroptera, respectively. For bats, echolocation status [2] is indicated by superscripts, numbered as follows: 1 = brief broadband tongue clicks (no laryngeal echolocation), 2 = no echolocation, 3 = constant frequency, 4 = short, broadband, multiharmonic, 5 = narrowband, multiharmonic, 6 = narrowband, dominated by fundamental harmonic, 7 = short, broadband, dominated by fundamental harmonic. Residue numbers are based on the human FOXP2 orthologue. Amino acids that were linked to the evolution of language are given in red and those only found in bats are given in blue. (0.18 MB DOC) [file pone.0000900.s002.doc]

|  | | species | | 6 | 11 | 22 | 41 | 42 | 43 | 46 | 47 | 65 | 71 | 78 | 79 | 80 | 84 | 87 | 99 | 197 | 244 | 250 | 256 | 266 | 278 | 279 | 280 | 283 | 284 | 294 | 298 | 302 | 303 | 304 |
| --- | --- | --- | --- | --- | --- | --- | --- | --- | --- | --- | --- | --- | --- | --- | --- | --- | --- | --- | --- | --- | --- | --- | --- | --- | --- | --- | --- | --- | --- | --- | --- | --- | --- | --- |
| S | O | consensus | | A | S | T | S | S | E | T | V | L | S | S | S | D | P | V | I | A | I | A | L | I | S | M | E | G | I | N | S | S | T | T |
| E | Pr | human | | . | . | . | . | . | . | . | . | . | . | . | . | . | . | . | . | . | . | . | . | . | . | . | . | . | . | . | . | . | N | . |
|  |  | gorilla, chimp, gibbon, macaque, baboon | | . | . | . | . | . | . | . | . | . | . | . | . | . | . | . | . | . | . | . | . | . | . | . | . | . | . | . | . | . | . | . |
|  |  | orang utan | | V | . | . | . | . | . | . | . | . | . | . | . | . | . | . | . | . | . | . | . | . | . | . | . | . | . | . | . | . | . | . |
|  |  | common marmoset | | . | . | . | . | . | . | . | . | . | . | . | . | . | . | . | . | . | . | . | . | . | . | . | . | . | . | . | . | . | . | . |
|  |  | galago | | . | . | . | . | . | . | . | . | . | . | N | . | . | . | . | . | . | . | . | . | . | . | . | . | . | . | . | . | . | . | . |
|  |  | gray mouse lemur | | . | . | . | . | . | . | . | . | . | . | . | . | . | . | . | . | V | . | . | . | . | . | . | . | . | . | . | . | . | . | . |
|  | Eu | Oriental water shrew | | . | . | . | . | . | . | . | . | I | . | . | . | . | . | . | . | . | L | . | . | . | . | . | . | A | . | . | . | . | . | . |
|  |  | Eurasian shrew | | . | . | . | . | . | . | . | . | . | . | . | . | . | . | . | . | . | L | . | . | . | . | . | . | A | . | . | . | . | . | . |
|  |  | African hedgehog | | . | . | . | . | . | . | . | . | . | . | N | . | E | . | . | . | . | . | T | . | . | . | . | . | . | . | . | . | . | . | . |
|  | R | mouse | | . | . | . | . | . | . | . | . | . | . | . | . | E | . | . | . | . | . | . | . | . | . | . | . | . | . | . | . | . | . | . |
|  | L | rabbit | | . | . | . | . | . | . | . | . | . | . | . | . | . | . | . | M | . | . | . | . | . | . | . | . | . | . | . | . | . | . | . |
| A | X | nine-banded armadillo | | . | . | . | . | . | . | . | . | . | . | . | . | . | . | . | . | . | . | . | . | . | . | . | . | . | . | . | . | . | . | . |
|  | Pr | African elephant | | . | . | . | . | . | . | . | . | . | . | . | . | . | . | . | . | . | . | . | . | . | . | . | . | . | . | . | . | . | . | . |
| L | Ar | pig | | . | . | . | . | . | . | . | . | . | . | . | . | . | . | . | . | . | . | . | . | . | . | . | D | . | . | . | . | . | . | . |
|  |  | goat | | . | . | . | . | . | . | . | . | . | . | . | . | . | . | . | . | . | . | . | . | . | . | . | . | . | . | . | . | . | . | . |
|  | Pe | donkey | | . | . | . | . | . | . | . | . | . | . | . | G | . | . | . | . | . | . | . | . | . | . | . | . | . | . | . | . | . | . | . |
|  | Ca | hog badger | | . | . | A | . | . | D | P | . | . | . | . | . | . | . | . | . | . | . | . | . | . | . | . | . | . | . | . | . | . | . | . |
|  |  | cat | | . | . |  | . | .. | . | . | . | . | . | . | . | . | . | . | . | . | . | . | . | . | . | . | . | . | . | . | . | . | . | . |
|  | Ch | P (Yi) | *Rousettus leschenaulti*^1^ | . | . | . | . | . | . | . | . | . | . | . | . | . | . | . | . | . | . | . | . | . | . | . | . | . | . | . | . | . | . | . |
|  |  | P (Yi) | *Cynopterus sphinx*^2^ | . | . | . | . | . | . | . | . | . | . | **G** | . | . | . | . | . | . | . | **G** | . | . | . | . | . | . | . | . | . | . | . | **I** |
|  |  | R (Yi) | *Rhinolophus ferrumequinun*^3^ | . | . | . | . | . | . | . | . | . | . | . | . | . | . | . | . | . | . | . | . | . | . | . | . | . | . | . | . | . | . | . |
|  |  | R (Yi) | *Rhinolophus luctus*^3^ | . | **T** | . | . | . | . | . | . | . | . | . | . | . | . | . | . | . | . | . | . | . | . | . | . | . | . | . | . | . | . | . |
|  |  | H (Yi) | *Aselliscus stoliczkanus*^3^ | . | **T** | . | . | . | . | . | . | . | . | . | . | . | . | . | . | . | . | . | . | . | . | . | . | . | . | . | . | . | . | . |
|  |  | H (Yi) | *Coelops frithi*^3^ | . | **T** | . | . | . | . | . | . | . | . | . | . | . | **A** | . | . | . | . | . | . | **L** | . | . | . | . | . | **S** | . | . | . | . |
|  |  | H (Yi) | *Hipposideros armiger*^3^ | . | **T** | . | . | . | . | . | . | . | . | . | . | . | . | . | . | . | . | . | **M** | . | . | . | . | . | . | . | . | . | . | . |
|  |  | Me (Yi) | *Megaderma spasma^4^* | . | . | A |  | **A** | . | P | **A** | . | . | . | T | . | . | . | . | V | . | . | . | . | **N** | . | . | . | . | **T** | . | . | **S** | . |
|  |  | E (Ya) | Taphozous melanopogon^5^ | . | . | . | . | . | . | . | . | . | **G** | . | . | . | . | . | . | . | . | . | . | . | . | **L** | . | . | . | . | . | . | . | . |
|  |  | M (Ya) | Chaerephon plicata^6^ | . | . | . | . | **T** | . | . | . | I | . | . | . | . | . | . | . | . | . | . | . | . | . | . | . | . | . | . | **L** | . | . | . |
|  |  | V (Ya) | *Miniopterus schreibersi*^6^ | . | . | . | . | . | . | . | . | . | . | . | . | . | . | **I** | . | V | . | . | . | . | **N** | . | . | . | . | . | **L** | . | . | . |
|  |  | V (Ya*)* | *Myotis ricketti*^7^ | . | . | . | **N** | . | . | . | **A** | . | . | . | . | . | . | . | . | V | . | . | . | . | . | . | . | . | . | . | **L** | **P** | . | . |
|  |  | V (Ya) | *Tylonycteris pachypus*^6^ | . | . | . | . | . | . | . | . | . | . | . | . | . | . | . | . | V | . | . | . | . | . | . | . | . | **M** | . | **L** | **P** |  | . |
