# Supplementary material for: Accelerated FoxP2 Evolution in Echolocating Bats
Source: PLoS One. 2007 Sep 19;2(9):e900. doi: 10.1371/journal.pone.0000900 (PMC1976393; doi:10.1371/journal.pone.0000900)
Supplement: Table S3 — FoxP2 gene (second half) with variable sites shown. For abbreviations, see Table S2. (0.17 MB DOC) [file pone.0000900.s003.doc]

|  |  | species | | 307 | 309 | 312 | 315 | 316 | 317 | 322 | 323 | 325 | 326 | 333 | 336 | 339 | 434 | 457 | 461 | 471 | 632 | 635 | 643 | 652 | 666 | 671 | 679 | 687 | 691 | 694 | 705 | 707 | 713 |
| --- | --- | --- | --- | --- | --- | --- | --- | --- | --- | --- | --- | --- | --- | --- | --- | --- | --- | --- | --- | --- | --- | --- | --- | --- | --- | --- | --- | --- | --- | --- | --- | --- | --- |
| S | O | consensus | | A | P | T | S | I | V | S | V | N | A | H | T | S | N | I | P | N | N | S | E | I | P | I | I | M | T | N | I | E | D |
| E | Pr | human | | . | . | . | . | . | . | . | . | S | . | . | . | . | . | . | . | . | . | . | . | . | . | . | . | . | . | . | . | . | . |
|  |  | gorilla, chimp, gibbon, macaque, baboon | | . | . | . | . | . | . | . | . | . | . | . | . | . | . | . | . | . | . | . | . | . | . | . | . | . | . | . | . | . | . |
|  |  | Orang utan | | . | . | . | . | . | . | . | . | . | . | . | . | . | . | . | . | . | . | . | . | . | . | . | . | . | . | . | . | . | . |
|  |  | common marmoset | | . | . | . | . | . | . | . | . | . | . | . | . | . | . | . | . | . | . | . | . | M | . | . | . | . | . | . | . | . | . |
|  |  | galago | | . | . | . | . | . | . | . | . | . | . | . | . | . | . | . | . | S | G | . | . | . | . | . | . | . | . | . | . | . | . |
|  |  | gray mouse lemur | | . | . | . | . | . | . | . | . | . | . | . | . | . | . | . | . | . | . | . | . | . | . | . | . | . | . | . | . | . | . |
|  | Eu | Oriental water shrew | | . | . | . | . | . | . | . | . | . | . | . | . | . | . | . | . | . | . | . | . | . | . | . | . | . | . | . | . | . | . |
|  |  | Eurasian shrew | | . | . | . | . | . | . | . | . | . | . | . | . | . | . | . | . | . | . | . | . | . | . | . | . | . | . | . | . | . | . |
|  |  | African hedgehog | | . | . | . | . | . | . | . | . | . | S | . | . | . | . | . | . | . | . | . | . | . | . | . | . | . | . | . | . | . | . |
|  | R | mouse | | . | . | . | . | . | . | . | . | . | . | . | . | . | . | . | . | . | . | . | . | . | . | . | . | . | . | . | . | . | . |
|  | L | rabbit | | . | . | . | . | . | . | . | . | . | . | . | . | . | . | . | . | . | . | . | . | . | . | . | . | . | . | . | . | . | . |
| A | X | nine-banded armadillo | | . | . | . | . | . | . | . | . | . | . | . | . | . | . | . | . | . | . | . | . | . | . | . | . | . | . | . | . | . | . |
|  | Pr | African elephant | | . | . | . | . | . | . | . | . | . | . | . | . | C | . | . | . | . | . | . | . | . | . | . | . | . | . | . | . | . | . |
| L | Ar | pig | | . | . | . | . | . | . | . | . | .. |  | . | . | . | . | . | . | . | . | . | . | L | . | . | M | . | . | . | . | . | . |
|  |  | goat | | . | . | . | T | . | . | . | . | . | . | . | . | . | . | . | . | . | . | . | . | . | . | . | . | . | . | . | . | . | . |
|  | Pe | donkey | | . | . | . | . | . | . | . | . | . | . | . | . | . | . | . | . | . | . | . | . | . | . | . | . | . | . | . | . | . | . |
|  | Ca | hog badger | | . | . | . | . | . | . | . | . | **S** | . | . | . | . | . | . | . | . | . | . | . | . | . | . | . | . | . | . | . | . | . |
|  |  | cat | | . | . | . | . | . | . | . | . | **S** | . | . | . | . | . | . | . | . | . | . | . | . | . | . | . | . | . | . | . | . | . |
|  | Ch | P (Yi) | *Rousettus leschenaulti­*1 | **V** | . | . | . | . | . | . | **L** | . | . | . | . | . | . | . | . | . | . | . | . | . | . | . | . | . | . | . | . | . | . |
|  |  | P (Yi) | *Cynopterus sphinx*2 | **V** | . | . | . | . | **M** | . | **H** | . | . | . | . | . | . | . | . | . | . | . | . | . | **S** | . | . | . | . | . | . | . | . |
|  |  | R (Yi) | *Rhinolophus ferrumequinum*3 | . | . | **P** | . | . | . | **A** | . | **S** | . | . | **P** | . | . | **L** | **A** | . | . | . | . | . | . | . | . | . | **A** | . | . | . | **E** |
|  |  | R (Yi) | *Rhinolophus luctus*3 | . | . | **P** | . | . | . | **A** | . | **S** | . | . | **P** | . | . | **L** | **A** | . | . | . | . | . | . | . | . | . | **A** | . | . | . | **E** |
|  |  | H (Yi) | *Aselliscus stoliczkanus*3 | . | . | **P** | . | **M** | . | . | . | **S** | . | . | . | . | . | **L** | **A** | . | . | . | . | M | . | . | . | **I** | . | . | . | **D** | **E** |
|  |  | H (Yi) | *Coelops frithi*3 | . | . | **P** | . | **M** | . | . | . | **S** | . | . | . | . | . | **L** | **A** | . | . | . | . | . | . | **V** | . | **I** | . | **D** | **M** | **D** | **E** |
|  |  | H (Yi) | *Hipposideros armiger*3 | . | . | **P** | . | **M** | . | . | . | **S** | . | . | . | . | **T** | **L** | **A** | . | . | . | . | . | . | . | . | **I** | . | . | . | . | **E** |
|  |  | Me (Yi) | *Megaderma spasma4* |  |  |  |  |  |  |  |  | **S** |  | **L** | . | . | **.** | **L** | . | . | . | . | **D** | M | . | . | . | . | . | . | . | . | . |
|  |  | E (Ya) | Taphozous melanopogon5 | . | . | . | . | . | . | . | . | . | . | . | **P** | . | . | . | . | . | . | . | . | . | . | . | . | . | . | . | . | . | . |
|  |  | M (Ya) | Chaerephon plicata6 | **V** |  | . | . | . | . | . | . | . | . | . | . | . | . | . | . | . | . | . | . | . | . | . | . | **V** | . | . | . | . | . |
|  |  | V (Ya) | *Miniopterus schreibersi*5 | . | **S** | . | . | . | . | . | . | . | . | . | . | . | . | . | . | . | . | . | . | . | . | . | . | **V** | . | . | . | . | . |
|  |  | V (Ya*)* | *Myotis ricketti*7 | . | . | . | . | . | . | . | . | . | **T** | . | . | . | . | . | . | . | . | . |  | M | . | . | M | **V** | . | . | . | . | . |
|  |  | V (Ya) | *Tylonycteris pachypus*6 | . | . | . | . | . | . | . | . |  | **T** | . | . | . | . | . | . | . | **T** | . |  | M | . | . | . | **V** | . | . | . | . | . |
