# Supplementary material for: Accelerated FoxP2 Evolution in Echolocating Bats
Source: PLoS One. 2007 Sep 19;2(9):e900. doi: 10.1371/journal.pone.0000900 (PMC1976393; doi:10.1371/journal.pone.0000900)
Supplement: Table S4 — Exon 7 of FoxP2. For abbreviations, see Table S2. (0.13 MB DOC) [file pone.0000900.s004.doc]

|  |  | species | | 278 | 279 | 280 | 283 | 284 | 294 | 298 | 302 | 303 | 304 | 307 | 309 | 312 | 315 | 316 | 317 | 321 | 322 | 323 | 325 | 326 |
| --- | --- | --- | --- | --- | --- | --- | --- | --- | --- | --- | --- | --- | --- | --- | --- | --- | --- | --- | --- | --- | --- | --- | --- | --- |
| S | O | consensus | | S | M | E | G | I | N | S | S | T | T | A | P | T | S | I | V | S | S | V | N | A |
| E | Pr | human | | . | . | . | . | . | . | . | . | N | . | . | . | . | . | . | . | . | . | . | S | . |
|  |  | 5 apes, 3 monkeys, lemur and galago | | . | . | . | . | . | . | . | . | . | . | . | . | . | . | . | . | . | . | . | . | . |
|  | Eu | Eurasian shrew and Oriental water shrew | | . | . | . | A | . | . | . | . | . | . | . | . | . | . | . | . | . | . | . | . | . |
|  |  | African hedgehog | | . | . | . | . | . | . | . | . | . | . | . | . | . | . | . | . | . | . | . | . | S |
|  | R/L | mouse and rabbit | | . | . | . | . | . | . | . | . | . | . | . | . | . | . | . | . | . | . | . | . | . |
| Af | X | nine-banded armadillo | | . | . | . | . | . | . | . | . | . | . | . | . | . | . | . | . | . | . | . | . | . |
|  | M/Pr | elephant shrew and African elephant | | . | . | . | . | . | . | . | . | . | . | . | . | . | . | . | . | . | . | . | . | . |
| L | Ce | 18 cetaceans | | . | . | . | . | . | . | . | P | . | A | . | . | . | . | M | . | . | . | . | . | . |
|  | Ar | goat | | . | . | . | . | . | . | . | . | . | . | . | . | . | T | . | . | . | . | . | . | . |
|  |  | pig | | . | . | D | . | . | . | . | . | . | . | . | . | . | . | . | . | . | . | . | . | . |
|  | Pe | donkey | | . | . | . | . | . | . | . | . | . | . | . | . | . | . | . | . | . | . | . | . | . |
|  | Ca | hog badger and cat | | . | . | . | . | . | . | . | . | . | . | . | . | . | . | . | . | . | . | . | **S** | . |
|  | Ch | P (Yi) | *Eonycteris spelaea*^2^, *Pteropus rodricensis*^2^ and *Rousettus leschenaulti*^1^ | . | . | . | . | . | . | . | . | . | . | **V** | . | . | . | . | . | . | . | **L** | . | . |
|  |  | P (Yi) | Cynopterus sphinx^2^ | . | . | . | . | . | . | . | . | . | **I** | **V** | . | . | . | . | **M** | . | . | **H** | . | . |
|  |  | R (Yi) | *Rhinolophus ferrumequinum*^3^, *R. luctus*^3^, *R. marshalli* ^3^and *R.paradoxolophus*^3^ | . | . | . | . | . | . | . | . | . | . | . | . | **P** | . | . | . | . | **A** | . | **S** | . |
|  |  | R (Yi) | Rhinolophus pusillus^3^ | . | . | . | . | . | . | . | . | . | . | . | . | **P** | . | . | . | **A** | **A** | . | **S** | . |
|  |  | H (Yi) | *Aselliscus stoliczkanus*^3^, *A. tricuspidatus*^3^*,* *Hipposideros amiger*^3^, *H. larvatus*^3^ and *H. pomona*^3^ | . | . | . | . | . | . | . | . | . | . | . | . | **P** | . | M | . | . | . | . | **S** | . |
|  |  | H (Yi) | Coelops frithi^3^ | . | . | . | . | . | **S** | . | . | . | . | . | . | **P** | . | M | . | . | . | . | **S** | . |
|  |  | Me (Yi) | *Megaderma spasma*^4^ and *M. lyra*^4^ | **N** | . | . | . | . | **T** | . | . | **S** | . | . | . | . | . | . | . | . | . | . | **S** | . |
|  |  | E (Ya) | Taphozous melanopogon^5^ | . | **L** | . | . | . | . | . | . | . | . | . | . | . | . | . | . | . | . | . | . | . |
|  |  | Ph (Ya) | Carollia perspicillata^4^ | . | . | . | . | **M** | . | **L** | . | . | . | . | . | . | . | . | . | **T** | . | . | **S** | . |
|  |  | Mo (Ya) | *Pteronotus macleayii*^5^, *P. parnellii*^3^ and *P*. *quadridens*^5^ | . | . | . | . | **M** | . | . | . | . | . | . | . | . | . | . | . | . | . | . | **T** | . |
|  |  | V (Ya) | Tylonycteris pachypus^6^ | . | . | . | . | **M** | . | **L** | P | . | . | . | . | . | . | . | . | . | . | . | . | **T** |
|  |  | V (Ya) | *Barbastella leucomelas*^7^, *Ia io*^6^, *Murina sp*^7^, *Myotis ricketti*^7^, *Pipistrellus abramus*^6^, *Plecotus sp*.^4^, *Scotomanes ornatus*^6^ and *Scotophilus kuhlii*^6^ | . | . | . | . | . | . | **L** | P | . | . | . | . | . | . | . | . | . | . | . | . | **T** |
|  |  | M (Ya) | Chaerephon plicata^6^ | . | . | . | . | . | . | **L** | . | . | . | **V** | . | . | . | . | . | . | . | . | . | . |
|  |  | V (Ya) | Miniopterus schreibersi^6^ | **N** | . | . | . | . | . | **L** | . | . | . | . | **S** | . | . | . | . | . | . | . | . | . |
