# Supplementary material for: Accelerated FoxP2 Evolution in Echolocating Bats
Source: PLoS One. 2007 Sep 19;2(9):e900. doi: 10.1371/journal.pone.0000900 (PMC1976393; doi:10.1371/journal.pone.0000900)
Supplement: Table S5 — Exon 17 of FoxP2. For abbreviations, see Table S2. (0.10 MB DOC) [file pone.0000900.s005.doc]

|  |  | species | | 671 | 678 | 679 | 680 | 687 | 691 | 692 | 694 | 698 | 699 | 701 | 705 | 707 | 710 | 713 |
| --- | --- | --- | --- | --- | --- | --- | --- | --- | --- | --- | --- | --- | --- | --- | --- | --- | --- | --- |
| S | O | consensus | | I | V | I | A | M | T | T | N | E | L | D | I | E | L | D |
| E | Pr | human | | . | . | . | . | . | . | . | . | . | . | . | . | . | . | . |
|  |  | 5 apes, 3 monkeys, lemur and galago | | . | . | . | . | . | . | . | . | . | . | . | . | . | . | . |
|  | Eu | Eurasian shrew, Oriental water shrew and African hedgehog | | . | . | . | . | . | . | . | . | . | . | . | . | . | . | . |
|  | R/L | mouse, rabbit, and flying squirrel | | . | . | . | . | . | . | . | . | . | . | . | . | . | . | . |
| Af | X | nine-banded armadillo | | . | . | . | . | . | . | . | . | . | . | . | . | . | . | . |
|  | M/Pr | elephant shrew and African elephant | | . | . | . | . | . | . | . | . | . | . | . | . | . | . | . |
| L | Ce | 15 cetaceans | | . | . | . |  | . | . | . | . | . | . | . | . | . | . | . |
|  | Ar/Pe | goat donkey and horse | | . | . | . | . | . | . | . | . | . | . | . | . | . | . | . |
|  |  | pig | | . | . | M | . | . | . | . | . | . | . | . | . | . | . | . |
|  | Ca | hog badger and cat | | . | . | . | . | . | . | . | . | . | . | . | . | . | . | . |
|  | Ch | P (Yi) | *Cynopterus sphinx^2^*, *Eonycteris spelaea^2^*, *Nyctimene cephalotes^2^*, *Pteropus rodricensis^2^* and *Rousettus leschenaulti^1^* | . | . | . | . | . | . | . | . | . | . | . | . | . | . | . |
|  |  | R (Yi) | *Rhinolophus affinis^3^*, *R. ferrumequinum^3^*, *R. luctus^3^*, *R*. *macrotis^3^*, *R. marshalli^3^*, *R. osgoodi^3^*, *R. pearsonii^3^*, *R. pusillus^3^* and *R. paradoxolophus^3^* | . | . | . | . | . | **A** | . | . | . | . | . | . | . | . | **E** |
|  |  | R (Yi) | *Aselliscus stoliczkanus^3^* and *A. tricuspidatus^3^* | . | . | . | . | **I** | . | . | . | . | . | . | . | **D** | . | **E** |
|  |  | H (Yi) | Hipposideros amiger^3^, H. larvatus^3^, H. pomona^3^ and H. pratti^3^ | . | . | . | . | **I** | . | . | . | . | . | . | . | . | . | **E** |
|  |  | H (Yi) | Coelops frithi^3^ | **V** | . | . | . | **I** | . | . | **D** | . | . | . | **M** | **D** | . | **E** |
|  |  | Me (Yi) | Megaderma spasma^4^ |  |  |  |  |  |  |  |  |  |  |  |  |  |  |  |
|  |  | E (Ya) | Taphozous melanopogon^5^ | . | . | . | . | . | . | . | . | . | . | . | . | . | . | . |
|  |  | N (Ya) | Nycteris tragata^4^ | . | **A** | . | **T** | . | . | **A** | . | **A** | **F** | **E** | **V** | . | **S** | . |
|  |  | Ph (Ya) | Carollia perspicillata^4^ | . | . | . | . | . | . | . | . | . | . | . | . | . | . | . |
|  |  | Mo (Ya) | *Mormoops blainvillii*^5^, *Pteronotus macleayii*^5^, and *P*. *quadridens*^5^ | . | . | . | . | . | . | . | . | . | . | . | . | . | . | . |
|  |  | Mo (Ya) | P. parnellii^3^ | . | . | . | . | **I** | . | . | . | . | . | . | . | . | . | . |
|  |  | V (Ya) | *Barbastella leucomelas*^7^, *Ia io*^6^, *Miniopterus schreibersi*^6^, *Nyctalus velutinus*^6^, *Plecotus sp*.^4^, *Scotomanes ornatus*^6^, *Scotophilus kuhlii*^6^*,* *Tylonycteris pachypus*^6^ and *Vespertilio sinensis*^6^ | . | . | . | . | **V** | . | . | . | . | . | . | . | . | . | . |
|  |  | V (Ya) | Murina sp.^7^ and Myotis ricketti^7^ | . | . | M | . | **V** | . | . | . | . | . | . | . | . | . | . |
|  |  | M (Ya) | Chaerephon plicata^6^ | . | . | . | . | **V** | . | . | . | . | . | . | . | . | . | . |
