# Supplementary figures and images for: Accelerated FoxP2 Evolution in Echolocating Bats
Source: PLoS One. 2007 Sep 19;2(9):e900. doi: 10.1371/journal.pone.0000900 (PMC1976393; doi:10.1371/journal.pone.0000900)

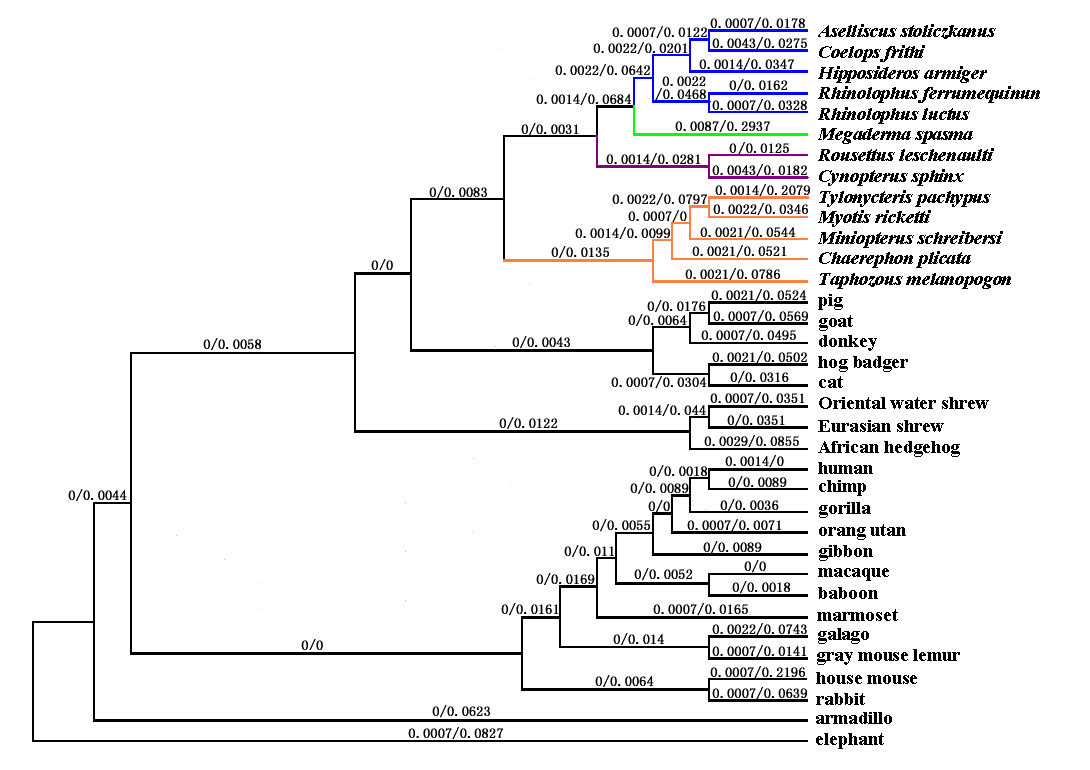

Supplement: Figure S1 — Cladogram showing PAML-derived maximum likelihood estimates of rates of non-synonymous and synonymous substitutions. Bat lineages are coloured to show the two major divergent forms of laryngeal echolocation (blue = high duty constant frequency (CF) calls with at least partial Doppler shift compensation, orange = low duty cycle calls, green = short, broadband multi-harmonic, violet = the absence of laryngeal echolocation in fruit bats. Estimates of the number of non-synonymous substitutions (dN) and synonymous substitutions (dS) under a free ratio model are given on the branches as (dN/dS). (0.05 MB DOC) [file pone.0000900.s006.doc]
